# Konjunktivektomie, Amnionmembrantransplantation als Multilayer-Graft und systemische Immunsuppression bei bilateraler peripherer ulzeröser Keratitis

**DOI:** 10.1007/s00347-020-01305-0

**Published:** 2021-01-05

**Authors:** S. Razafimino, I. Marjanovic, E. Flockerzi, B. Seitz

**Affiliations:** Klinik für Augenheilkunde, Universitätsklinikum des Saarlandes (UKS), Gebäude 22, Kirrberger Str. 100, 66424 Homburg/Saar, Deutschland

## Anamnese

Im April 2018 stellte sich eine 38-jährige kaukasische Patientin erstmals notfallmäßig in unserer Klinik vor. Die Patientin berichtete über seit 1 Monat bestehende Augenschmerzen, Rötung, Epiphora und Photophobie links mehr als rechts sowie Visusminderung am linken Auge. Eine 10 Tage zuvor eingeleitete Therapie mit Prednisolonacetat 1 % Augentropfen stündlich erbrachte keine wesentliche Befundbesserung. Anamnestisch bestanden keine ophthalmologischen Vorerkrankungen. In der allgemeinen Anamnese wurden von der Patientin bis auf kürzliche Zeichen einer atopischen Dermatitis keine weiteren Erkrankungen angegeben. Die Familienanamnese war ebenfalls leer.

## Klinischer Befund

**Figure 1:**
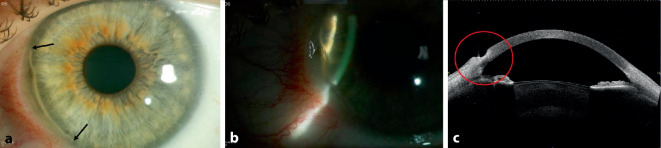

Bei der Erstvorstellung betrug die bestkorrigierte Sehschärfe 1,0 am rechten Auge und 0,6 am linken Auge. Der Augeninnendruck war im Normbereich (16 mm Hg beidseits). Der ophthalmologische Befund zeigte links mehr als rechts eine asymmetrische bilaterale Randfurchenkeratitis von 7 bis 9 Uhr, die limbusparallel halbmondförmig verlief. Die periphere Hornhaut zeigte sich ulzeriert, aber ohne Hornhautinfiltration oder intraokulare Entzündungszeichen. Die Bindehaut zeigte sich im befallenen Bereich injiziert (Abb. 1 und 2).

**Figure 2:**
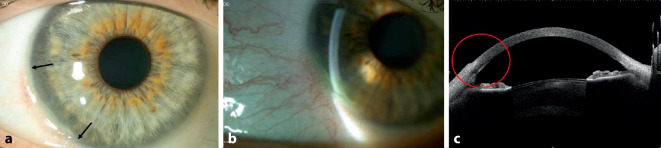

Die Untersuchung ergab keinen Hinweis auf eine assoziierte Skleritis, Limbusstammzellinsuffizienz, Lidfehlstellung, Blepharitis oder schwere Meibom-Drüsen-Dysfunktion. Fundoskopisch zeigte sich beidseits ein Normalbefund. Rheumatologische Erkrankungen wie eine rheumatoide Arthritis, ein Lupus erythematodes, die Sarkoidose, weitere Kollagenosen, die Granulomatose mit Polyangiitis und Morbus Behçet wurden anamnetisch und labormedizinisch ausgeschlossen. Die Blutuntersuchung umfasste ein vollständiges Blutbild mit CRP, Leber‑, Nierenwerten und Serologie auf Rheumafaktoren, antinukleäre Antikörper (ANA) mit Anti-Ro und Anti-SSA, Anti-Neutrophilen-Zytoplasma-Antikörper (ANCA), Angiotensin-Converting-Enzym (ACE), HLA B51, HLA B27, Anti-Doppelstrang-DNA-Antikörper, Antikörper gegen zyklische citrullinierte Peptide (CCP) und den löslichen Interleukin-2-Rezeptor. Nur der HLA B27-Test erwies sich als positiv. Hepatitis-B- und -C-Serologie sowie die Antikörperbestimmung gegen Toxoplasmose waren negativ. Eine Thoraxröntgenaufnahme zum Ausschluss einer Sarkoidose oder einer Tuberkulose wurde noch vor Einleitung einer systemischen Kortison-Therapie durchgeführt und zeigte sich unauffällig.

## Therapie und Verlauf

**Figure 3:**
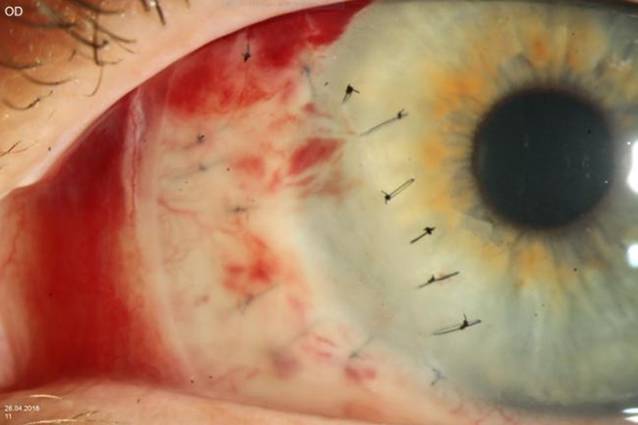

Die Patientin wurde stationär aufgenommen und mit systemischem Methylprednisolon (150 mg) mit schleichender Reduzierung über 6 Wochen und mit lokalen Prednisolonacetat-Augentropfen 1 %ig 3‑mal täglich und lokalen Ofloxacin-Augentropfen 5‑mal täglich an beiden Augen behandelt. Außerdem wurde zur Behandlung der atopischen Dermatitis eine Antikörperbehandlung mit Dupilumab subkutan (600 mg als Anfangsdosis, gefolgt von 300 mg alle 2 Wochen) eingeleitet. Es handelt sich um einen neuartigen humanen monoklonalen Antikörper, der auf die Interleukine 4 (IL-4) und 13 (IL-13) abzielt, 2 wichtige Zytokine, die bei atopischer Dermatitis eine pathologische Rolle spielen. Bei deutlichem Hornhautsubstanzverlust und Perforationsrisiko am linken Auge wurde eine Amnionmembrantransplantation (AMT) als Double-Graft mit konjunktivaler Exzision durchgeführt (Abb. 3). Nach Konjunktivektomie und vollständiger Entfernung des Epithels wurden 2 Amnionmembranschichten mit mehreren Einzelknüpfnähten im Ulkus fixiert (die Anzahl der Schichten ist abhängig von der Tiefe der Läsion). Die Konjunktivektomie soll Immunkomplexe entfernen, die Produktion von Kollagenasen und Proteinasen senken und damit die Auflösung der lokalen Entzündung fördern. Eine Verbandskontaktlinse wurde eingesetzt und nach 1 Monat wieder entfernt.

**Figure 4:**
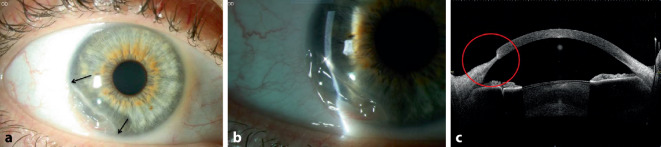

Der postoperative Verlauf gestaltete sich komplikationslos. Vier Monate später stellte sich die Patientin wegen Befundverschlechterung mit deutlichem Substanzverlust am rechten Auge vor mit jedoch noch bestkorrigiertem Visus von 0,8 (Abb. 4), sodass die gleiche Operation mit Amnionmembrantransplantation als Triple-Graft am rechten Auge durchgeführt wurde.

Zudem wurde eine systemische immunsuppressive Therapie mit Cyclosporin A 100 mg 2‑mal täglich und lokalen Cyclosporin-Augentropfen 1 %ig 1‑mal täglich hinzugefügt. Die AMT-Fäden wurden an beiden Augen 4 Wochen postoperativ entfernt.

**Figure 5:**
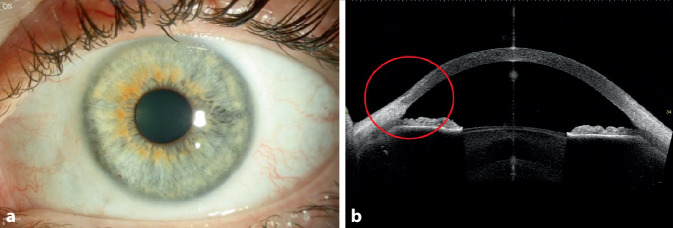

Die klinischen Ergebnisse 1, 2, 12 und 24 Monate postoperativ zeigten einen stabilen Befund, keine Entzündungszeichen, eine vollständige Abheilung des Geschwürs, keinerlei Rezidivzeichen. Die bestkorrigierte Sehschärfe blieb am rechten Auge stabil (0,8) und verbesserte sich am linken Auge (von 0,6 präoperativ auf 0,8 nach 24 Monaten) (Abb. 5 und 6).

**Figure 6:**
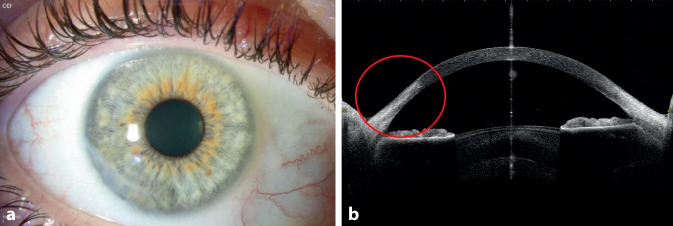

## Diskussion

Die periphere ulzeröse Keratitis (PUK) ist eine Form der Randfurchenkeratitis, die die juxtalimbale Hornhaut betrifft und durch eine sektorielle Ausdünnung des betroffenen Bereichs charakterisiert ist. Sie ist immer mit einem Epitheldefekt und einem fortschreitenden Verlust des Hornhautstromas verbunden. Die PUK ist oft zusammenhängend mit einer benachbarten konjunktivalen, episkleralen und skleralen Entzündung. Die PUK kann durch provozierten Astigmatismus und Hornhauttrübungen rasch zu einem Visusverlust und im schlimmsten Fall zu einer Hornhautperforation führen. Assoziierte Komplikationen können durch frühzeitige Diagnose, Erkennung der zugrunde liegenden systemischen Entzündungserkrankung und geeignete Behandlung vermieden werden [5].

Die Pathogenese der PUK ist noch nicht vollständig geklärt, aber sowohl T‑Zell- als auch antikörpervermittelte Wege sind in den Krankheitsprozess involviert [3]. Es wird vermutet, dass abnorme T‑Zellen zur Antikörperproduktion und zur Bildung von Immunkomplexen führen, die sich in der peripheren Hornhaut ablagern [6]. Der Komplementpfad wird durch die Rekrutierung von Entzündungszellen in die Hornhaut aktiviert. Kollagenasen und andere Proteasen werden von Neutrophilen und Makrophagen sezerniert, was zur Zerstörung des peripheren Hornhautstromas führt [3].

Da dies die erste Manifestation einer schweren, bisher unbekannten systemischen Erkrankung sein kann, ist eine rheumatologische und immunologische Abklärung dringend notwendig und erfordert eine interdisziplinäre Betreuung. In unserem Fall fand regelmäßig eine interdisziplinäre Rücksprache mit Rheumatologen/Immunologen und Dermatologen statt. Differenzialdiagnostisch müssen ein Ulcus Mooren, Morbus Fuchs-Terrien und eine pellucide marginale Degeneration einbezogen werden. Okuläre und systemische Infektionen können ebenfalls eine PUK verursachen oder mit einer PUK assoziiert sein. In unserem Fall wurde beidseits eine lokale Infektionsursache (bakteriell, viral, mykotisch) durch die mikrobiologische Untersuchung des entnommenen Bindehautgewebes ausgeschlossen.

Das Management der PUK sollte ein schrittweises Vorgehen entsprechend dem Schweregrad der Erkrankung enthalten. Es umfasst – in dieser Reihenfolge – in der Regel die lokale und systemische Kortikosteroidtherapie, die systemische Immunsuppression in den schwersten Fällen [9], gefolgt von zusätzlichen chirurgischen Eingriffen wie die konjunktivale Exzision, Amnionmembrantransplantation, lamelläre und perforierende Keratoplastik oder Cyanacrylat-Klebstoff bei Perforation [4, 7].

Chen et al. bewiesen die Effektivität der lamellären Keratektomie in Kombination mit einer lokalen Anwendung von Cyclosporin A, um die anatomische Integrität der Hornhaut und eine gute Sehschärfe zu erhalten [2].

Die konjunktivale Exzision ermöglicht die Entfernung einer großen Anzahl von Zellen und Entzündungsfaktoren in einem einzigen Block, was zu einer Abnahme der Kollagenaseproduktion führt und damit die Entzündungsreaktion lokal reduziert. Die Stromaverdünnung persistiert jedoch lokal, und es kann zu Rezidiven der Ulzeration kommen [10].

Die AMT als Multilayer-Graft [1] reduziert die Entzündung, beschleunigt aber auch die Reepithelialisierung und bedeckt das Ulkus, wodurch eine Perforation vermieden und die Integrität der Hornhaut erhalten wird [4, 8]. Bei unserer Patientin zeigte sich eine AMT als Multilayer-Graft in Kombination mit einer konjunktivalen Exzision als eine geeignete Methode, um die korneale Stabilität durch Integration der Amnionmembranen in das Hornhautstroma wiederherzustellen [1, 11]. Die lokale Entzündungsreaktion wurde reduziert, die Epithelialisierung beschleunigt, ein guter Visus konnte erzielt und erhalten werden und invasivere chirurgische Optionen wie eine perforierende Keratoplastik konnten vermieden werden.

## Fazit für die Praxis

- Bei einer PUK ist es wichtig, zunächst eine systemische Ätiologie auszuschließen.
- Um die Prognose zu verbessern, ist ein schnelles Management essenziell.
- Die Amnionmembrantransplantation als Multilayer-Graft in Kombination mit einer lokalisierten Konjunktivektomie scheint ideal, um die Integrität der Hornhaut zu erhalten.
- Um eine Progression zu vermeiden, ist die frühzeitige Einleitung einer systemischen Immunsuppression unerlässlich.
